# Patterns and factors among oncology fellows recommending medical cannabis to adults with cancer

**DOI:** 10.1186/s42238-025-00293-9

**Published:** 2025-07-14

**Authors:** Kian Tehranchi, Rushad Patell, Poorva Bindal, Laura Dodge, Jason Freed, Mary Buss, Mara A. Schonberg, Ilana Braun, Deepa Rangachari

**Affiliations:** 1 Department of Medicine, Beth Israel Deaconess Medical Center, Boston, MA USA; 2 Division of Hematology and Hematologic Malignancies, Beth Israel Deaconess Medical Center, Boston, MA USA; 3 Division of Hematology and Oncology, University of Massachusetts Memorial Health, Worcester, MA USA; 4 Department of Obstetrics and Gynecology, Beth Israel Deaconess Medical Center, Harvard Medical School, Boston, MA USA; 5 Department of Epidemiology, Harvard T.H. Chan School of Public Health, Boston, MA USA; 6 Palliative Care Services, Department of Medicine, UConn Health, University of Connecticut School of Medicine, Farmington, CT USA; 7 Department of Psychosocial Oncology and Palliative Care, Dana-Faber Cancer Institute, Boston, MA USA; 8 Department of Psychiatry, Harvard Medical School, Boston, MA USA; 9 Division of Medical Oncology, Beth Israel Deaconess Medical Center, Harvard Medical School, 330 Brookline Ave, 02215 Boston, MA USA

**Keywords:** Cannabis, Oncology, Education, Fellows

## Abstract

**Background:**

Medical cannabis consumption is rising, but limited evidence informs the safety and efficacy of cannabis use in cancer patients. A national survey of oncology trainees found that most fellows felt insufficiently informed to make clinical recommendations about cannabis.

**Aim:**

In this secondary analysis, we aimed to measure how frequently trainees recommend in favor of cannabis and determine factors influencing this clinical practice.

**Methods:**

In this cross-sectional survey study for fellows enrolled in oncology training programs across the United States, an online survey assessing trainee practices regarding medical cannabis was sent to 155 oncology fellowship program directors from January - March 2021; who were asked to distribute it to their fellows. The primary outcome was the frequency with which oncology fellows recommended cannabis in the prior year.

**Results:**

Nationally, 40 programs from 25 states participated, with 189 of 462 trainees across these programs responding (40.9% response rate). 22% (95% CI: 16.3–29.0%) of participants reported recommending medical cannabis to > 5 patients in the past year. 24% (95% CI: 18.4–30.5%) of participants had prior training in medical cannabis. Regarding participant characteristics, only prior training in medical cannabis was significantly associated with recommending cannabis to > 5 patients (RR: 2.4; 95% CI: 1.4–4.2).

**Conclusions:**

With increasing cannabis use among patients with cancer and given that a substantial number of oncology fellows recommend its use, it is crucial that fellowship training incorporate evidence-based curricula regarding medical cannabis use to guide informed decision-making between patients and their fellow providers.

**Supplementary Information:**

The online version contains supplementary material available at 10.1186/s42238-025-00293-9.

## Introduction

Over the past decade, access to medical cannabis has increased in the United States, with 38 states now having legalized medical cannabis (Pergam et al. 2017). Medical cannabis use among patients with cancer is also becoming increasingly common, with literature suggesting that 20–40% or more of contemporary cancer patients are using medical cannabis for physical and psychological symptoms(Pergam et al. 2017; Weiss et al. 2022; McClure et al. 2023). Despite the growing popularity of medical cannabis among adults with cancer, there is a lack of high-quality evidence to support its use in this population (Wang et al. 2008; Evanoff et al. 2017; St Pierre et al. 2020; Ellison et al. 2024). While there are few randomized controlled clinical trials on medical cannabis in cancer patients, most studies on medical cannabis and cancer have been small, observational, or retrospective, making it difficult to draw definitive conclusions about its safety and efficacy (Lynch and Ware 2015; Johnson et al. 2010; Portenoy et al. 2012; Braun et al. 2024a, b).

Patients with cancer who are using medical cannabis report desiring guidance from clinicians regarding its use (Pergam et al. 2017; Ware 2016). Yet, structured training on medical cannabis is uncommon in hematology/oncology fellowship training programs in the United States (Patell et al. 2022). Recent national guidelines for the use of medical cannabis in oncology populations recommend open discussions between providers and patients with a focus on goals of use, potential toxicities and limit recommendations for symptom control to guideline concordant use such as antiemetic prophylaxis (Braun et al. 2024a, b). With little formal training and a lack of high-quality evidence on medical cannabis use amongst their patients, oncology trainees may be unprepared to manage this increasingly common aspect of their patients’ care. Understanding trainee practices and perspectives on recommending cannabis can thus inform the development of training during fellowship.

Thus in 2021, we conducted a national survey of oncology fellows to assess their practices regarding counseling patients on medical cannabis and their perceptions of its effectiveness and risks. In our primary analysis, we found that while the majority of oncology trainees reported engaging in discussions regarding medical cannabis, most fellows also felt insufficiently informed to guide clinical decision-making regarding medical cannabis (Patell et al. 2022). In this secondary analysis of that survey, we aimed to measure how frequently trainees recommend cannabis and determine factors that influence this practice. Given the lack of rigorous evidence supporting its use in cancer patients, we hypothesized that fellows who had received prior training in medical cannabis use would be less likely to recommend it to their patients. Further, we aimed to measure the correlation between a trainee having an articulated opinion on the effects of medical cannabis with having received prior training and recommendation rates of medical cannabis.

## Methods

This is a cross-sectional survey study of fellows enrolled in oncology training programs across the United States. The Institutional Review Board at the Beth Israel Deaconess Medical Center approved the study as exempt from human subjects’ research. The methodology for the primary analysis is described previously (Patell et al. 2022). In summary, between January 1, 2021, and March 1, 2021, a survey assessing oncology trainee perceptions on medical cannabis use among cancer patients was developed by a multi-disciplinary team with expertise in oncology, psycho-oncology, and qualitative methodology. The instrument was piloted on five trainees and then anonymously administered through a web-based platform, REDCap Survey (Research Electronic Data Capture)(Harris et al. 2019; Harris et al. 2009), to fellows enrolled in oncology training programs in the United States. (Supplement) In order to distribute the survey, contact details of program directors (PDs) of all American College of Graduate Medical Education-accredited hematology/oncology fellowship training programs were obtained. The survey invitation was electronically sent to all PDs (*n* = 155) accompanied by a letter requesting them to forward the survey to all the fellows in their programs. This included programs in states that did have legal cannabis access. Participating PDs affirmed their program’s participation electronically and provided the number of fellows in their program to whom the survey was forwarded. The survey was preceded by an information sheet outlining study details, including purpose, risks, benefits, and investigator contact information. Consent was implied by voluntary completion of the survey, consistent with standard practice for minimal-risk, anonymous survey research. No attempt was made by the authors to directly contact trainees in participating sites. Two reminder emails were sent one week apart to PDs. Trainees were incentivized to participate using $25 gift cards offered through a random lottery.

The survey items most relevant to this secondary analysis included the frequency with which oncology trainees recommended medical cannabis to their patients and whether they had any prior training in medical cannabis. Response categories for the frequency of recommending medical cannabis were grouped (e.g., 0–5, 6–10, 11–20, > 20 patients) rather than free-text responses for ease of completion of survey. Trainees were also queried on their perceptions of medical cannabis in the following four knowledge domains: (Pergam et al. 2017) effectiveness of medical cannabis compared to conventional care for cancer-related symptoms (e.g. vomiting, diarrhea, pain); (Weiss et al. 2022) risks of medical cannabis as compared to prescription opioids (e.g. paranoia, falls, lung injury); (McClure et al. 2023) oncology populations most benefiting from medical cannabis (e.g. patients with early stage disease, patients at end of life, cancer survivors, young/old patients with cancer); and (Wang et al. 2008) preferred mode of administration of medical cannabis for oncology patients (e.g. oral ingestion, vaporization, smoking). The survey also collected participants’ demographic information including age, sex, ethnicity, region of practice, and educational background. Results from other items within the survey have been reported in the primary analysis (Patell et al. 2022).

### Analysis

The primary outcome was the number of patients to whom each oncology trainee had recommended medical cannabis in the prior year. Given skew towards recommending medical cannabis to fewer patients, For the purposes of analysis these responses were further grouped into those participants recommending medical cannabis to at least 5 patients and those recommending cannabis to greater than 5 participants. Log-binomial regression was used to calculate risk ratios (RR) and 95% confidence intervals (CI) to evaluate whether the number of cannabis recommendations varied by participant age, gender, legality of cannabis in the state of practice, and prior training in medical cannabis. We considered p values of < 0.05 to be statistically significant. We performed all analyses using SAS, version 9.4 (SAS Institute, Cary, NC) and GraphPad Prism for Windows (GraphPad Software, La Jolla, CA).

Participants were categorized as having an “articulated opinion” based on their responses to the queries in each of the four knowledge domains. In knowledge domains pertaining to cannabis effectiveness, cannabis risks, and cancer populations assessed to be benefitting from medical cannabis use, participants had the option to rate their views on a favorability scale from “very favorable” to “very unfavorable,” with an option to mark “I don’t know.” Participants who responded with “I don’t know” responses to < 25% of questions in each category were considered as having an articulated opinion in that knowledge domain. With regards to the mode of cannabis use, participants who stated they had any preferred mode of use for their cancer patients were considered as having an articulated opinion. A chi-squared test was used to test for associations between having an articulated opinion and prior training and recommendation frequency.

## Results

### Participant characteristics

Among the 155 training programs contacted, 40 PDs from 25 states agreed to distribute the survey to their fellows (25.8% response rate). One hundred eighty-nine of the 462 trainees in those programs completed the survey (40.9% response rate). Of the 189 participants completing the survey, 98 (52.4%) identified as non-Hispanic White, 62 (33.2%) as non-Hispanic Asian, 10 (5.4%) as Hispanic, and 2 (1.1%) as non-Hispanic Black (Table 1); 97 (52.2%) of participants were female. 78% (*n* = 142) of participants reported attending medical school in the United States, 1% (*n* = 2) in Canada, and 20% (*n* = 37) outside the US/Canada. The majority of participants in this study were in fellowship training in the Northeast (35.5%, *n* = 66), followed by the South (31.2%, *n* = 58), Midwest (21.0%, *n* = 39), and West (12.4%, *n* = 23). With respect to the self-reported legal status of cannabis in the participants’ state of practice, 43.6% (*n* = 81) of participants worked in states with comprehensive medical laws only, while 37.1% (*n* = 69) of participants practiced in states with comprehensive medical and recreational laws.

**Table 1:** Participant characteristics

Characteristic	*All Participants N = 189* *n* (%)	Recommended Cannabis to 5 Patients or Less *n* = 148,* n(%)*	Recommended Cannabis to > 5 Patients *n* = 41,* n(%)*
Birth year			
* 1975–1984*	19 (10.2)	14 (9.6)	5 (12.5)
* 1985–1989*	118 (63.4)	90 (61.6)	28 (70.0)
* 1990–1994*	49 (26.3)	42 (28.8)	7 (17.5)
Year of medical school graduation			
* 2003–2015*	62 (33.7)	45 (31.0)	17 (43.6)
* 2015–2020*	122 (66.3)	100 (69.0)	22 (56.4)
Female	97 (52.2)	79 (54.1)	18 (45.0)
Race/ethnicity			
* White*,* non-Hispanic*	98 (52.4)	76 (51.7)	22 (55.0)
* Asian*	62 (33.2)	49 (33.3)	13 (32.5)
* Hispanic*	10 (5.4)	7 (4.8)	3 (7.5)
* Black or African-American*	2 (1.1)	2 (1.4)	0 (0.0)
* American Indian*	1 (0.5)	0 (0.0)	1 (2.5)
* More than one/other*	9 (4.8)	9 (6.1)	0 (0.0)
* Prefer not to answer*	5 (2.7)	4 (2.7)	1 (2.5)
Location of medical school			
* United States*	142 (78.5)	111 (78.2)	31 (79.5)
* Canada*	2 (1.1)	2 (1.4)	0 (0.0)
* Outside the US and Canada*	37 (20.4)	29 (20.4)	8 (20.5)
Training setting*			
* Academic hospital*	177 (93.7)	139 (93.9)	38 (92.7)
* VA hospital*	32 (16.9)	22 (14.9)	10 (24.4)
* Community-based practice*	14 (7.4)	11 (7.4)	3 (7.3)
* Other*	2 (1.1)	2 (1.4)	0 (0.0)
Census region of practice			
* Northeast*	66 (35.5)	50 (34.0)	16 (41.0)
* South*	58 (31.2)	51 (34.7)	7 (18.0)
* Midwest*	39 (21.0)	30 (20.4)	9 (23.1)
* West*	23 (12.4)	16 (10.9)	7 (18.0)
Legal status of cannabis in state of practice			
* Comprehensive medical and recreational*	69 (37.1)	54 (36.7)	15 (38.5)
* Comprehensive medical*	81 (43.6)	62 (42.2)	19 (48.7)
* Allows products with low THC/high CBD*	36 (19.4)	31 (21.1)	5 (12.8)
Area of focus*			
* Solid tumor oncology*	153 (81.0)	122 (82.4)	31 (75.6)
* Malignant hematology*	93 (49.2)	75 (50.7)	18 (43.9)
* Non-malignant hematology*	71 (37.6)	53 (35.8)	18 (43.9)
* Supportive/palliative care*	11 (5.8)	7 (4.7)	4 (9.8)
* Other*	4 (2.1)	4 (2.7)	0 (0.0)

*Participants could select more than one option, and thus responses may sum to more than 100%

### Opinions regarding modes of medical cannabis use

67% (*n* = 126) of participants listed oral ingestion as the recommended mode of medical cannabis use (Table 2), followed by vaporization (12.2%, *n* = 23), smoking (4.8%, *n* = 9) and rectal suppositories (2.7%, *n* = 5). 15% of participants (*n* = 29) stated they did not know what their preferred mode of use for adults with cancer would be, and 16.4% (*n* = 31) did not have a preference. The majority of participants (55.6%) reported that they did not have a preferred strain (also known as variety or chemovar) of medical cannabis for oncology patients.

**Table 2:** Preferred modes and strains

Characteristic	*N* = 189 *n* (%)
Mode of use for oncology patients*	
* Ingesting orally*	126 (66.7)
* Vaporizing*	23 (12.2)
* Smoking*	9 (4.8)
* Rectal suppository*	5 (2.7)
* No preference*	31 (16.4)
* Do not support medical cannabis use*	5 (2.7)
* Don’t know*	29 (15.5)
Preferred strain**	
* Rich in THC*	9 (4.8)
* Rich in CBD*	43 (22.8)
* Rich in both THC and CBD*	26 (13.8)
* Do not support medical cannabis use*	6 (3.2)
* Don’t know*	105 (55.6)

*Participants could select more than one option, and thus responses may sum to more than 100%

** preferred scientific terminology includes chemovar or variety

THC, tetrahydrocannabinol; CBD, cannabidiol

### Prior training regarding medical cannabis

24% (*n* = 46, 95% binomial CI: 18.4–30.5%) of participants had prior training in medical cannabis. Among the 46 participants who reported prior training, 47.8% (*n* = 22) reported that this training occurred during residency, 34.7% (*n* = 16) during fellowship, 26.0% (*n* = 12) through conferences, and 15.2% (*n* = 7) during medical school. With regards to format of training it included conferences (*n* = 13), webinars (*n* = 9).

### Patterns of recommending medical cannabis

22% (*n* = 148, 95% binomial CI: 16.3–29.0%) of participants reported recommending medical cannabis to > 5 patients in the past year, with 13.8% (*n* = 26) recommending to 6–10 patients, 5.3% (*n* = 10) to 11–20 patients, and 2.6% (*n* = 5) to > 21 patients. (Supplementary Table 1). With regards to participant characteristics, only prior training in medical cannabis was associated with recommending cannabis to > 5 patients (RR: 2.4; 95% CI: 1.4–4.2). None of the other variables including participants’ age, year of medical school graduation, gender, and legal status of cannabis in the state of training were associated with higher rates of recommending medical cannabis to patients (Table 3).

**Table 3:** Factors associated with recommending medical cannabis to more than five patients in the past year

Predictor	Unadjusted RR (95% CI)	Adjusted* RR (95% CI)
Birth year		
* 1975–1984*	1.84 (0.67–5.10)	--
* 1985–1989*	1.66 (0.78–3.55)	--
* 1990–1994*	1.00	--
**Year of medical school graduation**		
*** 2003–2015***	1.52 (0.87–2.65)	1.52 (0.89–2.59)
*** 2015–2020***	1.00	1.00
Female gender	0.77 (0.45–1.32)	--
US-based medical school	1.06 (0.53–2.13)	--
Prior training†		
* Academic hospital*	0.86 (0.31–2.38)	--
* VA hospital*	1.58 (0.87–2.89)	--
* Community-based practice*	0.99 (0.35–2.80)	--
* Other*	4.04 (0.96–17.03)	--
**Legal status in state of practice**		
*** Comprehensive medical + recreational***	1.00	1.00
*** Comprehensive medical***	1.08 (0.59–1.96)	1.05 (0.60–1.85)
*** Allows products with low THC/high CBD***	0.64 (0.25–1.62)	0.89 (0.34–2.32)
Area of focus†		
* Solid tumor oncology*	0.73 (0.40–1.35)	--
* Malignant hematology*	0.81 (0.47–1.40)	--
* Non-malignant hematology*	1.30 (0.76–2.23)	--
* Supportive/palliative care*	1.75 (0.76–4.02)	--
**Prior training in medical cannabis**	2.60 (1.54–4.40)	2.42 (1.39–4.20)

Data are shown as risk ratio (RR) and 95% confidence interval (CI)

*Adjusted for year of medical school graduation, legal status in state of practice, and having received prior training in medical cannabis (P for prior training: 0.002; all other variables *P* ≥ 0.13)

†Compared to not training or focusing in this area

### Opinions on knowledge domains in medical cannabis and associations with prior training and recommending patterns for medical cannabis

Prior training in medical cannabis was associated with an increased likelihood of having an articulated opinion in all four domains queried (effectiveness, side effects, populations benefiting, and preferred modes of administration) (Table 4). Participants were more likely to recommend medical cannabis to > 5 patients if they had an articulated opinion on the comparative effectiveness [RR 2.67 (1.4–5.3), *p* = 0.001] and preferred mode of administration [RR 3.15 (1.3–7.6), *p* = 0.01] of medical cannabis (Table 5). The association between having an articulated opinion on the side effects of medical cannabis and recommending medical cannabis to > 5 patients was not statistically significant (*p* = 0.10). There was no association between having an articulated opinion regarding subpopulations likely to benefit from medical cannabis and higher recommendations rates.

**Table 4:** Association between participant’s prior training in medical cannabis and having an articulated opinion in various knowledge domains

	Fellows with prior training in medical cannabis (*n* = 45)	Fellows without with prior training in medical cannabis (*n* = 143)	Risk Ratio (95% CI)	*p* value
**Effectiveness of medical cannabis for cancer related issues in comparison to conventional treatments**				
Responders with an Articulated Opinion*	36 (80.0)	71 (49.7)	1.61 (1.29–2.01)	< 0.001
**Side effects of medical cannabis compared to opioids**				
Responders with an Articulated Opinion*	42 (93.3)	109 (76.2)	1.22 (1.09–1.38)	0.01
**Sub-population likelihood to benefit from medical cannabis**				
Responders with an Articulated Opinion*	42 (93.3)	107 (74.8)	1.25 (1.10–1.41)	0.01
**Preferred mode of use**				
Responders with an Articulated Opinion*	39 (88.6)	88 (63.3)	1.40 (1.19–1.65)	< 0.001

Data are shown as n (%)

CI, confidence interval

*Participants had an “articulated opinion” if they answered at least 75% of questions in each knowledge domain. For cannabis effectiveness, risks, and benefits, they rated their views on a favorability scale. For the mode of use, indicating a preferred method qualified as an articulated opinion

**Table 5:** Association between participants having an articulated opinion in various knowledge domain and rates of recommending medical cannabis to patients

	Responders with an Articulated Opinion*	Responders without an Articulated Opinion	RR	*p* value
**Effectiveness of medical cannabis for cancer related issues in comparison to conventional treatments**				
Fellows who recommended medical cannabis to > 5 patients	32 (29.6)	9 (11.1)	2.67 (1.35–5.27)	0.001
**Side effects of medical cannabis compared to opioids**				
Fellows who recommended medical cannabis to > 5 patients	37 (24)	4 (11)	2.25 (0.86–5.92)	0.10
**Sub-population likelihood to benefit from medical cannabis**				
Fellows who recommended medical cannabis to > 5 patients	34 (23)	7 (18)	1.26 (0.61–2.63)	0.53
**Preferred mode of use**				
Fellows who recommended medical cannabis to > 5 patients	36 (28.1)	5 (8.9)	3.15 (1.31–7.60)	0.01

Data are shown as n (%)

*Participants had an “articulated opinion” if they answered at least 75% of questions in each knowledge domain. For cannabis effectiveness, risks, and benefits, they rated their views on a favorability scale. For the mode of use, indicating a preferred method qualified as an articulated opinion

Among these four domains, participants had the option to express how favorably versus unfavorably they viewed cannabis in the domains of effectiveness, side effects, and populations benefiting. In terms of the effectiveness of cannabis for cancer related issues, trained and untrained participants expressed closely split favorable and unfavorable views (Supplementary Tables 1–3). Both trained and untrained participants more often held unfavorable views on the side effects of medical cannabis. Alternatively, both trained and untrained participants more often held favorable views on the subpopulations of cancer patients potentially benefitting from medical cannabis.

## Discussion

### Main findings

Despite limited evidence to support medical cannabis use among adults with cancer, more than 1 in 5 oncology fellows participating in our study recommended it to > 5 patients in the past year. Approximately one quarter of respondents had received prior training, and these trainees were significantly more likely to have articulated opinions on modes of cannabis use, effectiveness, risks, and populations where medical cannabis may have utility. In this group, prior training was also associated with a doubling in the rates at which participants recommended medical cannabis to their patients.

### What this study adds

The results of this study build upon the primary analysis previously published by our group, demonstrating that more than half of oncology trainees reported discussing medical cannabis with > 5 patients in the past year (Patell et al. 2022). In sum, while more than half of trainees participating in our survey have *discussed* medical cannabis with a substantial number of their patients, fewer than half have translated these discussions into actual recommendations for cannabis use. In the primary analysis, prior training in medical cannabis was associated with a 50% increased likelihood of ‘discussing’ medical cannabis; the secondary analysis presented here further amplifies this and shows a 160% increased likelihood of *recommending* medical cannabis in the setting of prior training. This affirms the significant impact of curricula in medical cannabis use during medical training with regard to how often trainees feel prepared to discuss and/or recommend its use with patients. Given the limited high-quality evidence on medical cannabis use in oncology patients, these findings highlight the importance of formulating curricula using best available data on the subject to ensure well-informed patient-provider decision making while appreciating limitations in evidence (Braun et al. 2024a, b), in concordance with current national guidelines (Braun et al. 2024a, b). As trainees indicate that fellowship training is the most common time for this to occur (if at all), these findings point to an area of unmet educational need and an important opportunity to develop evidence-based curricula in this domain.

We found that the majority of participants in the study identified oral ingestion as their clinically preferred mode of medical cannabis use, followed by vaporization. The preference for oral ingestion may reflect both provider familiarity and the perceived safety and controllability of this route. Pharmacokinetic considerations such as bioavailability, timing of onset and duration of effect are tied to route of administration (Braun et al. 2024a, b). Current literature does not clearly endorse one route as superior across all clinical contexts, but in palliative and oncology care, the optimal route may vary depending on the symptom being targeted (Braun et al. 2024a, b; Steele et al. 2019). Interestingly, 15.5% of participants indicated they did not have knowledge on a preferred mode of use, and 16.4% had no preference. The heterogeneity in these responses may reflect that some providers lack awareness on the increasingly numerous available routes of administration, lack of robust safety data, and the absence of clinical guidelines to direct the safest and most effective use in cancer populations at the time of conducting the survey (Corroon et al. 2019). When comparing our results to those available in the published literature to date, practicing oncologists were more likely to indicate no preference or a lack of knowledge on a preferred mode of medical cannabis use (Sannes et al. 2021). Further, 15.4% of practicing oncologists reported not supporting medical cannabis use in their practice compared to just 2.7% of trainees in this study—again, recapitulating the notion that discussions surrounding cannabis use are increasing amongst younger generations of our work force (Jacobs et al. 2022; Weisman and Rodríguez 2021).

In this analysis, we found that prior training in medical cannabis was associated with having an articulated opinion on the comparative efficacy, risks, modes of use, and populations most likely to benefit from medical cannabis. However, in most cases training did not significantly influence whether those opinions were favorable or unfavorable. Interestingly, simply having an articulated view—particularly regarding efficacy and mode of use—was associated with higher rates of recommending medical cannabis. A similar, though nonsignificant, trend was observed for safety-related opinions, suggesting it may also be a critical domain to target in education. For instance, participants with an articulated opinion on cannabis efficacy recommended it more frequently, regardless of whether they viewed it positively or negatively. This underscores the importance of targeted education that promotes informed, nuanced understanding of cannabis in oncology care.

It is important to note that the evidence base for medical cannabis use in oncology varies by indication, with relatively stronger support for chemotherapy-induced nausea and vomiting, more limited data for chronic pain and cachexia, and little to no evidence for symptoms such as diarrhea (Braun et al. 2024a, b; Worster et al. 2022). Given the growing evidence on cannabis-related side effects and drug interactions, training should also equip fellows to assess safety risks and counsel patients appropriately, including those using cannabis recreationally (Braun et al. 2024a, b). This includes specific domains such as drug interactions, potential interactions with cancer directed therapy such as immunotherapy, hepatotoxicity and side effects (Nachnani et al. 2024; Woerdenbag et al. 2023). There is interest in the implementation of evidence based medical cannabis curriculum (Crowley et al. 2024). Attempts to do so during oncology fellowship training suggest this is both feasible and well received (Patell et al. 2025). Our results provide valuable insights regarding specific knowledge domains that may be most likely to impact recommendations for medical cannabis use and thus prioritized during the curriculum development process: comparative efficacy and modes of administration. It is critical for curricula designed for trainees in oncology thus highlight areas of certainty/uncertainty so as to facilitate truly informed patient-provider counseling and decision-making.

### Strengths and limitations

Our study’s strengths include the response rate and a robust sample size with strong representation of oncology trainees across the nation geographically. Our survey captured perspectives from trainees practicing in states with varying cannabis laws, enhancing the generalizability of our findings. The present study has limitations that should be taken into account. Similar to other online surveys, selection bias is possible as participants who chose to respond may have had stronger opinions about medical cannabis than the general population (Bethlehem 2010). Given the anonymous nature of the survey, we were unable to track non-respondents or collect detailed program-level data on cannabis legality or the presence of cannabis-specific curricula. As such, we cannot assess how these factors influenced response rates. As participants self-reported the legal status of cannabis in their respective states this could not thus be verified by the authors for each respondent. We acknowledge that self-reported data can be subject to inaccuracies due to factors such as recall bias or lack of awareness of state laws. Programs where formal training on medical cannabis is already incorporated may be more likely to distribute the survey to their trainees, potentially inflating the prevalence of structured training. We acknowledge that the source, quality, format, and timing of medical cannabis training may influence trainees’ knowledge. However, these details were not consistently captured in our dataset, and the limited sample size further constrained our ability to explore these factors. Future studies should aim to collect more granular data to better define emerging standards in medical cannabis education. As the policy landscape for medical cannabis evolves and research grows leading to increasing evidence regarding medical cannabis use in patients with cancer = z, future studies should re-examine oncology trainees’ knowledge, attitudes, and behaviors regarding its use periodically. New and existing curricula will need to be meticulously maintained to incorporate the most updated and rigorous evidence to keep up with the growing need (Zolotov et al. 2021). Future areas of investigation should explore how and when during training to best introduce evidence-based curricula on medical cannabis use and the impact of structured training in prospective studies, including assessments of durability of impact beyond training.

## Conclusion

Despite limited evidence to support medical cannabis use among cancer patients, 1 in 5 oncology fellows participating in our study recommended it to > 5 patients in the past year. Prior training in medical cannabis was the sole factor associated with higher rates of discussing and recommending its use to patients. Personalized, patient-centered care for cancer patients—and all patients—is mandatorily founded on understanding and articulating the best available evidence regarding treatment options. Accordingly, as medical cannabis gains more widespread legal status and is increasingly considered and used by our patients, it will be of critical importance that contemporary fellowship training programs incorporate rigorous, up-to-date curricula on this subject so as to prepare their trainees to engage in well-informed discussions and shared decision-making with those for whom they care.

## Electronic supplementary material

Below is the link to the electronic supplementary material.

Supplementary Material 1

Supplementary Material 2

## Data Availability

Data will be available by contacting the corresponding author on reasonable request.
