# Supplementary material for: Patterns and factors among oncology fellows recommending medical cannabis to adults with cancer
Source: J Cannabis Res. 2025 Jul 14;7:45. doi: 10.1186/s42238-025-00293-9 (PMC12261571; doi:10.1186/s42238-025-00293-9)
Supplement: Supplementary file 1 — Supplementary Material 1 [file 42238_2025_293_MOESM1_ESM.docx]

**Supplementary Tables**

**Supplement Table 1.** Participant rates of recommending medical cannabis

| **Number of patients in the past year for whom you recommended cannabis** | **N=189**  **n (%)** |
| --- | --- |
| *0-5* | 148 (78.3) |
| *6-10* | 26 (13.8) |
| *11-20* | 10 (5.3) |
| *>21* | 5 (2.6) |

**Supplementary Table 2.** Proportion of favorable versus unfavorable responses to queries on the effectiveness of medical cannabis for cancer related issues

|  | Unfavorable (% ± SD) | Neutral (% ± SD) | Favorable (% ± SD) | “I don’t know” responses (% ± SD) |
| --- | --- | --- | --- | --- |
| Prior training |  |  |  |  |
| *Yes (n=45)* | 30.6 ± 29.1 | 22.4 ± 24.1 | 31.8 ± 30.6 | 15.2 ± 30.7 |
| *No (n=143)* | 20.8 ± 28.7 | 18.8 ± 23.1 | 24.9 ± 29.0 | 35.5 ± 39.4 |
| *P value* | 0.05 | 0.37 | 0.18 | < 0.05 |
| “Articulated opinion”* |  |  |  |  |
| *Yes (n=120)* | 30.9 ± 30.4 | 25.1 ± 25.4 | 34.8 ± 30.8 | 9.2 ± 20.5 |
| *No (n=69)* | 10.8 ± 22.9 | 9.9 ± 15.4 | 11.8 ± 19.9 | 67.5 ± 33.8 |
| *P value* | < 0.05 | < 0.05 | < 0.05 | < 0.05 |

*Defined as being “more certain” on at least 3 domains (efficacy, risks, populations, modes)

**Supplementary Table 3.** Proportion of favorable versus unfavorable responses to queries on the side effects and risks of medical cannabis among patients with cancer

|  | Unfavorable (% ± SD) | Neutral (% ± SD) | Favorable (% ± SD) | “I don’t know” responses (% ± SD) |
| --- | --- | --- | --- | --- |
| Prior training |  |  |  |  |
| *Yes (n=45)* | 46.4 ± 25.4 | 28.1 ± 19.6 | 18.8 ± 17.4 | 6.7 ± 16.7 |
| *No (n=143)* | 39.4 ± 26.5 | 25.4 ± 22.2 | 16.2 ± 17.3 | 19.0 ± 30.3 |
| *P value* | 0.10 | 0.44 | 0.38 | <0.05 |
| “Articulated opinion”* |  |  |  |  |
| *Yes (n=120)* | 47.8 ± 25.5 | 30.3 ± 20.3 | 18.4 ± 16.8 | 3.5 ± 6.6 |
| *No (n=69)* | 29.7 ± 23.9 | 18.5 ± 21.9 | 14.0 ± 17.8 | 37.7 ± 36.7 |
| *P value* | <0.05 | <0.05 | 0.09 | < 0.05 |

*Defined as being “more certain” on at least 3 domains (efficacy, risks, populations, modes)

**Supplementary Table 4.** Proportion of favorable versus unfavorable responses to queries on how beneficial medical cannabis is for subpopulations of oncology patients

|  | Unfavorable (% ± SD) | Favorable (% ± SD) | “I don’t know” responses (% ± SD) |
| --- | --- | --- | --- |
| Prior training |  |  |  |
| *Yes (n=45)* | 19.2 ± 25.4 | 76.7 ± 29.4 | 4.2 ± 16.9 |
| *No (n=143)* | 16.7 ± 23.5 | 65.4 ± 33.3 | 17.9 ± 32.1 |
| *P value* | 0.55 | < 0.05 | < 0.05 |
| “Articulated opinion”* |  |  |  |
| *Yes (n=120)* | 19.4 ± 24.1 | 79.3 ± 24.8 | 1.3 ± 5.5 |
| *No (n=69)* | 13.4 ± 22.9 | 48.9 ± 35.9 | 37.7 ± 39.0 |
| *P value* | 0.09 | < 0.05 | < 0.05 |

*Defined as being “more certain” on at least 3 domains (efficacy, risks, populations, modes)
