# Supplementary material for: Patterns and factors among oncology fellows recommending medical cannabis to adults with cancer
Source: J Cannabis Res. 2025 Jul 14;7:45. doi: 10.1186/s42238-025-00293-9 (PMC12261571; doi:10.1186/s42238-025-00293-9)
Supplement: Supplementary file 2 — Supplementary Material 2 [file 42238_2025_293_MOESM2_ESM.pdf]

Medical Cannabis Survey

Rushad Patell and Poorva Bindal, Hem Onc Fellows at Beth Israel Deaconess Medical Center in Boston, are conducting a research study about the attitudes and practice around the use of medical marijuana in oncology patients. As a trainee in Oncology/Hematology or allied fields, you are invited to participate in this research study.

If you choose to be in the research study, you will complete the survey below. The survey will take about 10 minutes for you to complete.

You can skip any survey questions that you do not want to answer. Even if you start the survey, you are not required to complete it, partial responses will be saved. You can stop at any time. The survey is anonymous, and no one will be able to link your answers back to you. Please do not include your name or other information that could be used to identify you in the survey responses.

As we know how valuable your time is, we are offering ten $25 Amazon gift cards, that will be raffled at the end of the research study as an incentive. Include your email address if you are interested in the raffle, this will not be linked to your responses.

Being in this study is voluntary. Please contact Rushad Patell at rpatell@bidmc.harvard.edu or Poorva Bindal at pbindal@bidmc.harvard.edu with questions about this research study. If you have questions about your rights participating in research or would like to speak with someone independent from the research team, please contact the Human Subject Protection Office (617) 975-8500.

Thank you in advance for your time and participation.

Rushad and Poorva

USE OF MEDICAL CANNABIS IN PATIENTS WITH CANCER

Medical cannabis refers to non-pharmaceutical cannabis that healthcare providers recommends for therapeutic purposes. It does not refer to pharmaceutical-grade cannabinoids such as ingested synthetic THC analogs, dronabinol and nabilone

When you discuss medical cannabis with your oncology patients, who typically starts the conversation?

- ◯ More often I do
- ◯ Sometimes me and sometimes patients or their families
- ◯ More often patients or their families
- ◯ I have not discussed medical cannabis with my oncology patients or their families

In the past year, for approximately how many patients did you recommend cannabis use for cancer-related issues?

- ◯ 0-5
- ◯ 6-10
- ◯ 11-20
- ◯ 21-30
- ◯ 31-40
- ◯ >40

In the past year, for approximately how many patients did you discuss cannabis use for cancer-related issues?

- ◯ 0-5
- ◯ 6-10
- ◯ 11-20
- ◯ 21-30
- ◯ 31-40
- ◯ >40

Compared to treatment you typically use, how would you rate the effectiveness of medical cannabis for the following cancer related issues?

In your opinion, is medical cannabis:

	Much more effective	Somewhat more effective	Equally effective	Somewhat less effective	Much less effective	I don't know
Nausea/vomiting	◯	◯	◯	◯	◯	◯
Pain	◯	◯	◯	◯	◯	◯
Poor appetite/cachexia	◯	◯	◯	◯	◯	◯
Depression	◯	◯	◯	◯	◯	◯
Poor sleep	◯	◯	◯	◯	◯	◯
General coping	◯	◯	◯	◯	◯	◯
Anxiety	◯	◯	◯	◯	◯	◯

To what extent do you think medical cannabis has anti-neoplastic effects?

◯ To a great extent
◯ To some extent
◯ To a very small extent
◯ None at all
◯ I don't know

To what extent do you think medical cannabis is a useful adjunct to standard treatments for the following cancer related issues?

	To a great extent	To some extent	To a very small extent	Not at all
Pain	◯	◯	◯	◯
Anxiety	◯	◯	◯	◯

In your opinion, how do the risks of medical cannabis use compare to the risks of prescription opioid use?

The following risks of medical cannabis use are:

	Much higher than opioids	Somewhat higher than opioids	Comparable with opioids	Somewhat lower than opioids	Much lower than opioids	I don't know
Paranoia/psychosis	◯	◯	◯	◯	◯	◯
Anxiety	◯	◯	◯	◯	◯	◯
Depression	◯	◯	◯	◯	◯	◯
Confusion/impaired mentation	◯	◯	◯	◯	◯	◯
Falls	◯	◯	◯	◯	◯	◯
Driving difficulties	◯	◯	◯	◯	◯	◯
Lung injury	◯	◯	◯	◯	◯	◯
Cancer incidence or progression	◯	◯	◯	◯	◯	◯

Addiction	◯	◯	◯	◯	◯	◯
Overdose death	◯	◯	◯	◯	◯	◯

How concerned are you that smoking cannabis could increase the risk of infection in immunocompromised oncology patients ?

◯ Very concerned
◯ Somewhat concerned
◯ A little concerned
◯ Not at all concerned

In your opinion, how often is medical cannabis beneficial for the following types of oncology patients?

	Never beneficial	Rarely beneficial	Sometimes beneficial	Usually beneficial	Always beneficial	I don't know
Those near the end of life	◯	◯	◯	◯	◯	◯
Those with early stage cancer	◯	◯	◯	◯	◯	◯
Cancer survivors	◯	◯	◯	◯	◯	◯
Young adults with cancer	◯	◯	◯	◯	◯	◯
Elderly cancer patients	◯	◯	◯	◯	◯	◯
Those on active cancer treatment	◯	◯	◯	◯	◯	◯
Cannabis naive patients	◯	◯	◯	◯	◯	◯
Recreational cannabis users	◯	◯	◯	◯	◯	◯

What mode(s) of medical cannabis use do you prefer for your oncology patients ?
Mark all that apply

☐ Smoking
☐ Vaporizing
☐ Ingesting Orally
☐ Rectal suppository
☐ No preference
☐ I do not support medical cannabis use of any sort
☐ I don't know

Cannabis contains more than 60 active compounds. In general, do you prefer cannabis strains rich in tetrahydrocannabinol (THC) or cannabidiol (CBD) for oncology patients ?

◯ Rich in tetrahydrocannabinol (THC)
◯ Rich in cannabidiol (CBD)
◯ Rich in both
◯ I do not support medical cannabis use of any sort
◯ I don't know

Do you feel you have sufficient knowledge about medicinal use of cannabis to make recommendations for oncology patients?

◯ Yes
◯ No

From which of the following sources do you get the majority of your information on medical cannabis ?

◯ Peer-reviewed sources
◯ Lay media
◯ Cannabis dispensaries
◯ Patients and families
◯ Lecture/webinar by another physician
◯ Other sources

If other, please specify:

____

ABOUT YOU

In what year were you born?

____

How do you describe yourself?

- ◯ Male
- ◯ Female
- ◯ Other

Please indicate your race/ethnicity.

- ◯ African-american
- ◯ Asian
- ◯ Hispanic
- ◯ American Indian
- ◯ Pacific Islander
- ◯ White (non-hispanic)
- ◯ Other or combination
- ◯ Prefer not to answer

Have you received any training regarding medical cannabis?

- ◯ Yes
- ◯ No

If yes, then in what setting did you receive the training?
(select all that apply)

- ☐ College
- ☐ Medical School
- ☐ Residency
- ☐ Fellowship
- ☐ Webinars
- ☐ Conferences
- ☐ Other

If other, please specify:

____

What year did you graduate from medical school?

____

Where was the medical school located?

☐ USA ☐ Canada ☐ Another Country

Please specify:

____

What is your area of focus (select all that apply) ?

- ☐ Non-Malignant Hematology
- ☐ Malignant Hematology
- ☐ Solid Tumor Oncology
- ☐ Supportive/Palliative Care
- ☐ Other

If other, please specify:

____

In a typical week, approximately how many patients with cancer do you see?

- ◯ 0-5
- ◯ 6-10
- ◯ 11-15
- ◯ 16-20
- ◯ 21-25
- ◯ 26-30
- ◯ >30

In a typical week, how many hours do you spend directly seeing patients with cancer?	◯ 0-10<br>◯ 11-20<br>◯ 21-30<br>◯ 31-40<br>◯ 41-50<br>◯ 51-60<br>◯ >60
How would you best characterize the location of your training?	☐ Academic Hospital<br>☐ VA Hospital<br>☐ Community-based Practice<br>☐ Other
If other, please specify:	__________________________________

In which state is your training program located ?

- ◯ Alabama - AL
- ◯ Alaska - AK
- ◯ Arizona - AZ
- ◯ Arkansas - AR
- ◯ California - CA
- ◯ Colorado - CO
- ◯ Connecticut - CT
- ◯ Delaware - DE
- ◯ Florida - FL
- ◯ Georgia - GA
- ◯ Hawaii - HI
- ◯ Idaho - ID
- ◯ Illinois - IL
- ◯ Indiana - IN
- ◯ Iowa - IA
- ◯ Kansas - KS
- ◯ Kentucky - KY
- ◯ Louisiana - LA
- ◯ Maine - ME
- ◯ Maryland - MD
- ◯ Massachusetts - MA
- ◯ Michigan - MI
- ◯ Minnesota - MN
- ◯ Mississippi - MS
- ◯ Missouri - MO
- ◯ Montana - MT
- ◯ Nebraska - NE
- ◯ Nevada - NV
- ◯ New Hampshire - NH
- ◯ New Jersey - NJ
- ◯ New Mexico - NM
- ◯ New York - NY
- ◯ North Carolina - NC
- ◯ North Dakota - ND
- ◯ Ohio - OH
- ◯ Oklahoma - OK
- ◯ Oregon - OR
- ◯ Pennsylvania - PA
- ◯ Rhode Island - RI
- ◯ South Carolina - SC
- ◯ South Dakota - SD
- ◯ Tennessee - TN
- ◯ Texas - TX
- ◯ Utah - UT
- ◯ Vermont - VT
- ◯ Virginia - VA
- ◯ Washington - WA
- ◯ West Virginia - WV
- ◯ Wisconsin - WI
- ◯ Wyoming - WY
- ◯ Other

If other, please specify:

____

In the past year, for approximately how many oncology patients did you fill out paperwork allowing then to use medical cannabis in compliance with state law? ________________________________

(Medical cannabis refers to non-pharmaceutical cannabis that healthcare providers recommends for therapeutic purposes. It does not refer to pharmaceutical-grade cannabinoids such as ingested synthetic THC analogs, dronabinol and nabilone.)

Please let us know your thoughts on the use of medical cannabis for patients with cancer.

____

Please let us know your thoughts on what should be included in a training program for medical cannabis for patients with cancer. ____

If you would like to be entered into the raffle for a $25 gift cards, please enter your email address. ________________________________

(Answers will be kept anonymous)
